# Shifting attention between perception and working memory

**DOI:** 10.1016/j.cognition.2024.105731

**Published:** 2024-01-25

**Authors:** Daniela Gresch, Sage E.P. Boettcher, Freek van Ede, Anna C. Nobre

**Affiliations:** a Department of Experimental Psychology, University of Oxford, Oxford, UK; b Oxford Centre for Human Brain Activity, Wellcome Centre for Integrative Neuroimaging, Department of Psychiatry, University of Oxford, Oxford, UK; c Institute for Brain and Behavior Amsterdam, Department of Experimental and Applied Psychology, Vrije Universiteit Amsterdam, the Netherlands; d Wu Tsai Institute, Yale University, New Haven, CT, USA; e Department of Psychology, Yale University, New Haven, CT, USA

**Keywords:** Internal attention, External attention, Shifting attention, Fixational gaze behaviour, Vision

## Abstract

Most everyday tasks require shifting the focus of attention between sensory signals in the external environment and internal contents in working memory. To date, shifts of attention have been investigated within each domain, but shifts between the external and internal domain remain poorly understood. We developed a combined perception and working-memory task to investigate and compare the consequences of shifting spatial attention within and between domains in the service of a common orientation-reproduction task. Participants were sequentially cued to attend to items either in working memory or to an upcoming sensory stimulation. Stay trials provided a baseline condition, while shift trials required participants to shift their attention to another item within the same or different domain. Validating our experimental approach, we found evidence that participants shifted attention effectively in either domain (Experiment 1). In addition, we observed greater costs when transitioning attention between as compared to within domains (Experiments 1, 2). Strikingly, these costs persisted even when participants were given more time to complete the attentional shift (Experiment 2). Biases in fixational gaze behaviour tracked attentional orienting in both domains, but revealed no latency or magnitude difference for within- fversus between-domain shifts (Experiment 1). Collectively, the results from Experiments 1 and 2 suggest that shifting between attentional domains might be regulated by a unique control function. Our results break new ground for exploring the ubiquitous act of shifting attention between perception and working memory to guide adaptive behaviour in everyday cognition.

## 1 Introduction

Efficient goal-directed behaviour necessitates attention to be dynamically and selectively directed towards task-relevant contents available in the external world as well as those maintained in mind as internal representations (Narhi-Martinez, Dube, & Golomb, 2023; van Ede & Nobre, 2023). Accordingly, attention can be classified based on the domain in which it operates – either perception or memory (i.e., external attention or internal attention; Chun, Golomb, & Turk-Browne, 2011).

In laboratory tasks, pre-cues and retro-cues have emerged as valuable tools for studying attentional orienting mechanisms in relation to both perceptual and mnemonic contents, respectively. Orienting towards external information has been extensively investigated in tasks using predictive pre-cues that proactively provide information concerning a target-defining attribute prior to a target array (Posner, 1980). Conversely, tasks using retro-cues have proven effective for investigating internal orienting of attention to selective contents within working memory. Predictive retro-cues presented during the retention interval retroactively guide attention according to a target-defining attribute, such as its encoded location (Griffin & Nobre, 2003; Landman, Spekreijse, & Lamme, 2003). These lines of research have provided important insights into the limits, mechanisms, and associated neural systems of attentional orienting to perception and memory (Carrasco, 2011; Chun et al., 2011; Corbetta & Shulman, 2002; Gazzaley & Nobre, 2012; Kiyonaga & Egner, 2013; Lepsien & Nobre, 2006; Myers, Stokes, & Nobre, 2017; Nobre et al., 2004; Panichello & Buschman, 2021; Souza & Oberauer, 2016; Wallis, Stokes, Cousijn, Woolrich, & Nobre, 2015; Zhou, Curtis, Sreenivasan, & Fougnie, 2022). For instance, pre-cue and retro-cue studies have consistently demonstrated that selectively attending to perceptual or mnemonic task-relevant information results in improved behavioural outcomes and enhanced neural processing for the prioritised information.

Yet, surprisingly, the focus of these previous works has been almost entirely on how we orient and reorient attention *within* either perception or working memory. Outside of the laboratory, we often shift attention dynamically *between* contents in the environment and in our mind. For instance, a cyclist entering a busy intersection needs to attend to sensory contents, such as changing traffic lights and pedestrians crossing the street, as well as focus on mnemonic contents, such as memorised directions. Thus, the cyclist’s focus of attention must be in constant flux, rapidly transitioning between information available in the external environment and internal traces stored in memory. In this manner, adaptive behaviour within an ever-changing world calls for our ability to seamlessly shift attention between contents in perception and memory.

To date, a limited number of studies have investigated the flexible transitions of attentional selection between perception and memory. Existing work has emphasised that shifting between external and internal attentional domains may incur higher costs than reorienting within either domain. These studies have employed variations of task-switching paradigms, in which participants alternate between phases that require responding to sensory stimuli and phases that necessitate responding to encoded memoranda. Early behavioural studies used list-reproduction tasks requiring verbal or manual reports of digits or letter series which were either perceptually available, stored in memory, or required integration across both domains (Carlson, Wenger, & Sullivan, 1993; Dark, 1990; Weber, Burt, & Noll, 1986). After a relative hiatus, the question of shifting attention between sensory and internally generated information has attracted renewed interest (Calzolari, Boneva, & Fernández-Espejo, 2022; Gilbert, Frith, & Burgess, 2005; Hautekiet, Verschooren, Langerock, & Vergauwe, 2023; Honey, Newman, & Schapiro, 2017; Poskanzer & Aly, 2023; Servais, Hurter, & Barbeau, 2023; Steel, Silson, Garcia, & Robertson, 2024; Treder et al., 2021; Verschooren, Liefooghe, Brass, & Pourtois, 2019; Verschooren, Pourtois, & Egner, 2020; Verschooren, Schindler, De Raedt, & Pourtois, 2019; Verschooren, Schindler, De Raedt, & Pourtois, 2021; Weilnhammer, Stuke, Standvoss, & Sterzer, 2023). For instance, researchers have replicated earlier work within the visual domain using tasks that require participants to shift between perception and memory on a trial-by-trial basis (Hautekiet et al., 2023; Verschooren et al., 2020; Verschooren, Liefooghe, et al., 2019). In these studies, the external trial required comparing stimuli that were both shown on the screen simultaneously, while the internal trial involved retrieving a stimulus from memory and comparing it to a target on the screen. This line of research takes an important step towards understanding attentional flexibility. However, to fully capture the dynamic nature of attentional shifting that occurs in our daily lives, we must go further.

Two central issues remain to be addressed. The first issue is the need to capture the everyday situation of smoothly shifting between the contents available in the external world and our minds to perform a single task, without necessarily being required to respond to each of these contents. Thus far, paradigms inspired by task-switching approaches have been used to calculate costs of shifting between domains. For example, recent research has employed match-to-sample tasks in which participants responded either to externally presented or internally represented targets, with all other task requirements held constant across both attentional domains (Hautekiet et al., 2023; Verschooren et al., 2020; Verschooren, Liefooghe, et al., 2019). In these studies, participants responded according to either sensory information or memory representations on a per-trial basis. The cost of shifting between domains was revealed by reduced performance in subsequent trials of the alternate domain, as compared to those in the same domain. In contrast, in our daily lives, it is common to transition between external and internal attention in service of a unified goal-directed action. Returning to the example of the cyclist; taking a left turn at the traffic light involves multiple shifts between perception and memory (i.e., from externally attending to the traffic light, to internally attending to the memorised locations, to externally attending to the children crossing the street, etc.) before performing the single intended manual action (i.e., taking a turn).

The second important issue to address is how attention shifts between visual perception and visual working memory. Working memory is the core internal buffer for guiding adaptive behaviour (Funahashi, Bruce, & Goldman-Rakic, 1989; Fuster & Alexander, 1971; Myers et al., 2017; Nobre & Stokes, 2019; van Ede & Nobre, 2023). Yet, most previous studies have focused on attentional shifting between external and other internal domains, such as imagination (Gilbert et al., 2005) or self-oriented cognition (Calzolari et al., 2022). Even when internal attention was operationalised as memory, it was often limited to the verbal modality (Carlson et al., 1993; Dark, 1990; Weber, Burt, & Noll, 1986) or closely linked to long-term memory representations (Hautekiet et al., 2023; Verschooren et al., 2020, 2021; Verschooren, Liefooghe, et al., 2019; Verschooren, Schindler, et al., 2019).

The present two experiments build on the promising foundation from task-switching and attentional (re-)orienting literature to investigate how we shift attention within and between contents in visual perception and visual working memory to guide performance towards unified actions. We developed a double-cueing task using both pre-cues and retrocues in which participants had to orient and then reorient the focus of attention to external and internal stimuli within trials. Importantly, participants were presented with the same stimulus material and response demands across both attentional domains. On a behavioural level, our task allowed for all four possible directions of shifts to be tested, that is, external-to-external, internal-to-internal, internal-to-external, and external-to-internal – of which the first two engage within-domain shifts, while the latter two engage between-domain shifts.

In the first experiment we recorded gaze position as an implicit continuous proxy of spatial attention shifts as they unfolded, to supplement accuracy and response-time measures obtained at the end of the trials. Recent research has shown that small directional eye movements track the orienting of attention to memorised visual locations (Draschkow, Nobre, & van Ede, 2022; van Ede, Chekroud, & Nobre, 2019; van Ede, Deden, & Nobre, 2021), similar to orienting to external locations (Engbert & Kliegl, 2003; Hafed, Lovejoy, & Krauzlis, 2011). By leveraging this sensitive and time-resolved metric, we could compare the dynamics of attentional orienting to visual sensory and working-memory locations and of shifting within and between domains. In the second experiment, we included multiple intervals between the final attention-orienting cue and the response probe to gain further evidence regarding the time courses of shifting attention within versus between domains.

We predicted we would observe additional costs for shifting between as opposed to within domains in line with the previous literature (Calzolari et al., 2022; Carlson et al., 1993; Dark, 1990; Gilbert et al., 2005; Hautekiet et al., 2023; Verschooren et al., 2020; Verschooren, Liefooghe, et al., 2019; Weber, Burt, & Noll, 1986). However, we made no directional prediction concerning whether shifts would be faster or incur greater costs when moving from one specific domain to the other (i.e., internal-to-external vs. external-to-internal). Previous work has highlighted that shifting from the internal to the external domain may incur less costs than vice versa (Calzolari et al., 2022; Dark, 1990; Hautekiet et al., 2023; Verschooren et al., 2020). However, given the differences in our experimental design – specifically investigating attentional shifts towards a single response – we chose to remain neutral to the possible outcomes. Inclusion of gaze biases was intended to reveal any differences in temporal dynamics between shift types and to shed light on whether the timing of spatial shifts ultimately determined or impacted the final behavioural performance measure. We were mindful that performance differences could arise depending on whether participants responded to external or internal targets. Therefore, our analysis only compared responses to targets within a particular domain, albeit as a function of whether attention was initially oriented within that domain or to information in the alternate domain.

As a preview of our results, both Experiments 1 and 2 showed significantly higher response-time and accuracy costs after shifting attention between as compared to within domains. In both experiments, accuracy costs were only higher when shifting between relative to within domains if reporting an internal target, whereas no differences were detected whilst reporting an external target. Experiment 2 showed that between-domain shift costs remained consistent with intervals up to 750 ms after the final cue. The gaze results of Experiment 1 proved very interesting, showing similar timings for shifting attention within and between domains. The divergence in the timings of gaze shifts and reaction times at the end of the trial invite us to consider additional bottlenecks when shifting attention between domains, beyond just the attentional orienting phase.

## 2 Experiment 1

### 2.1 Methods

#### 2.1.1 Participants

The study was approved by the Central University Research Ethics Committee of the University of Oxford. The sample size was set to *n* = 25, based on previous working-memory studies from our lab with similar outcome measures (i.e., reaction times, reproduction errors, gaze biases; van Ede, Chekroud, & Nobre, 2019; van Ede, Deden, & Nobre, 2021). Additionally, we conducted a power analysis based on preliminary pilot data (*n* = 7). Employing the ‘mixedpower’ package (Kumle, Võ, & Draschkow, 2021) in RStudio (Posit team, 2022), we computed power simulations with 1000 iterations based on a model including both shift type and target domain as fixed effects. Given that our primary focus was on comparing different levels of shift type, we directed our attention towards this factor during the power-analysis interpretation. To account for the inherent uncertainty in effect size estimates from pilot data, we systematically varied the mean differences between conditions in order to perform more conservative power assessments. As illustrated in Supplementary Fig. 1A, with a sample size of *n* = 25, a 50% smaller effect in RTs would still provide power >80%.

To yield the targeted number of participants, we collected data from 32 participants in total. Four participants were excluded following our a-priori behavioural trial-removal procedure (see ‘Behavioural analysis’). In addition, three datasets were removed due to poor eye tracking (see ‘Eye-tracking analysis’ for further details). All remaining 25 participants (age range: 18 to 37; mean age: 25.48; gender: 12 female, 10 male, 3 non-binary; handedness: 21 right-handed, 4 left-handed) had normal or corrected-to-normal vision. Individuals provided written informed consent before participating in the study and were paid £15 per hour.

#### 2.1.2 Apparatus and data acquisition

Participants sat in front of a monitor (27-in. Acer XB270H; resolution 1920 × 1080 pixels; refresh rate 100 Hz; screen width 60 cm) at a viewing distance of 100 cm. An eye tracker (EyeLink 1000, SR Research) was positioned on the table approximately 15 cm in front of the monitor. Before acquisition, we calibrated the eye tracker using a 5-point calibration and validation method. Gaze was continuously tracked binocularly at a sampling rate of 1000 Hz, and participants were required to maintain fixation throughout the trial. The experimental script was generated in Python using the ‘PsychoPy’ package (version 2021.2.3; Peirce et al., 2019).

#### 2.1.3 Task and procedure

**Fig. 1:**
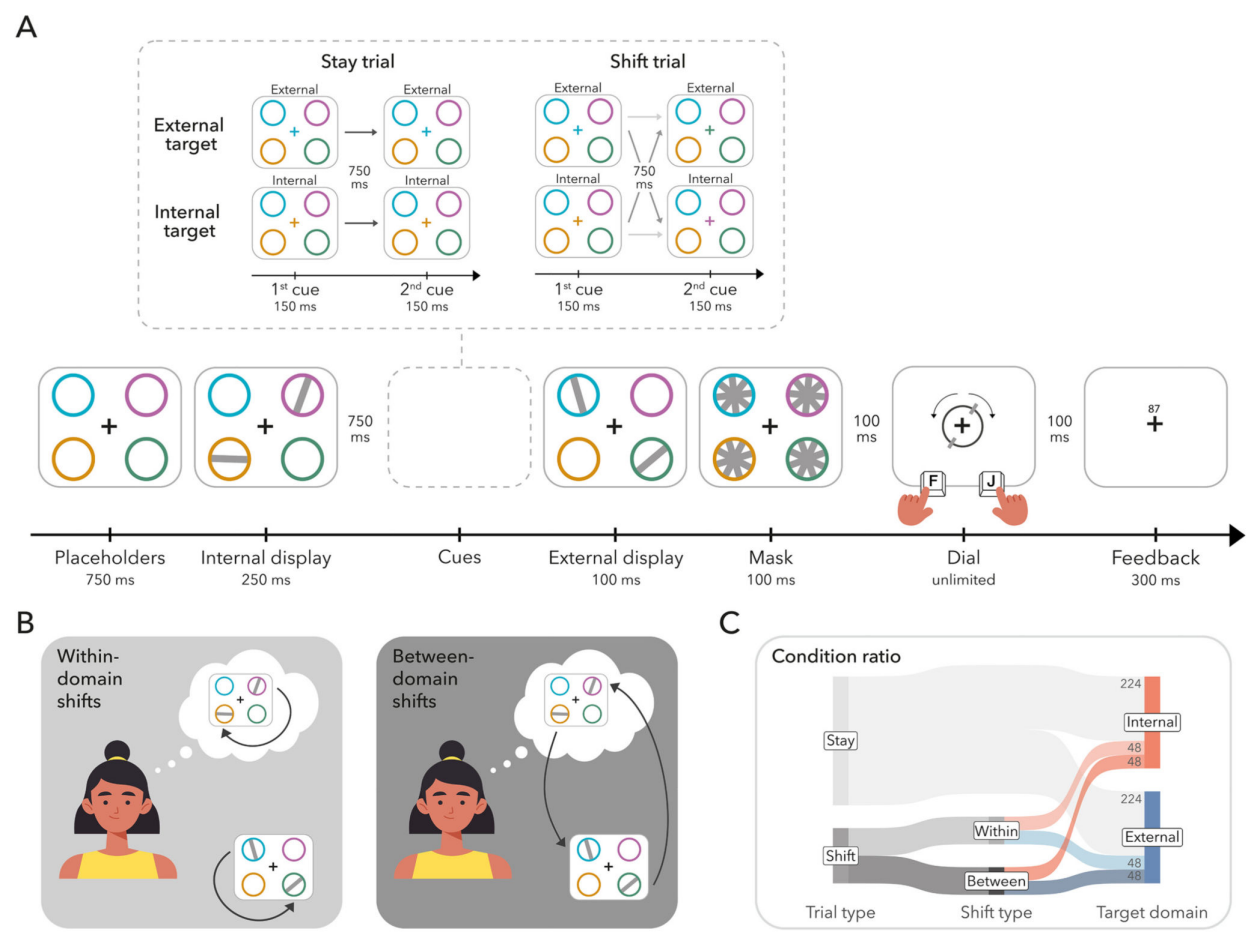
Task schematic and condition details of Experiment 1. (A) Illustration of stay and shift trials. In stay trials, the second cue indicated the same item as the first cue. In shift trials, the first and the second cue indicated different items. Light-grey arrows depict within-domain shifts and dark-grey arrows represent between-domain shifts. (B) In within-domain shift trials, participants either had to shift attention external-to-external or internal-to-internal. In between-domain shift trials, participants either had to shift attention external-to-internal or internal-to-external. (C) Ratio of conditions and number of trials across the experiment.

Participants performed a double-cueing task in which two separate bilateral arrays each containing randomly tilted bars were presented, with one occurring before and the the other after a sequential pair of spatially informative colour cues (Fig. 1A). At the end of each trial, participants reproduced the orientation of the last item to be cued. Importantly, items were presented across both an internal working-memory array, encoded before the cues, and an external perceptual array, appearing after the cues. As such, cues indicating the location of a previously encoded item shifted attention internally (i.e., retro-cues, internal cues), instructing which item in working memory would need to be reported. In contrast, cues indicating a previously unoccupied location shifted attention externally (i.e., pre-cues, external cues), instructing which item in the upcoming perceptual array would need to be reported.

The two main manipulations of this task were (1) that the first and second cue could either indicate the same or different items (stay vs. shift) and (2) that cued items could be upcoming sensory information or internal representations (external vs. internal).^1^ For shift trials, this meant that, at the time of the second cue, attention needed to be reoriented to either an item of the same domain (i.e., within-domain shift, Fig. 1B, left panel) or an item in the other domain (i.e., between-domain shift, Fig. 1B, right panel). This resulted in four possible shift conditions: external-to-external, internal-to-internal, internal-to-external, and external-to-internal, allowing us to compare the effect of shifting within and between attentional domains. In stay trials, the second-cued item was the same as the first-cued item. That is, both cues indicated either the same memory item or the same perceptual location. The second cue was 100% instructive, and thus, always indicated the target for later report. To ensure participants used the first cue, we made the first cue highly predictive for the later report by having disproportionally more stay trials (70%) relative to shift trials (30%). Fig. 1C illustrates the ratio of conditions and number of trials across the experiment.

At the start of each trial, a white central fixation cross (RGB value: [255, 255, 255]) surrounded by four coloured circles (i.e., placeholders) was presented against a black background (RGB value: [0,0,0]) for 750 ms. The colours of the placeholders in each trial were always randomly drawn from a set of four highly distinguishable colours (RGB values: blue [0,159,183], orange [217,120,0], green [47,167,0], pink [247,35,255]. The colour-location mapping was randomly determined on each trial. Placeholders subtended 3.5° visual angle (~195 pixels) and were centred in the top-left, top-right, bottom-left, and bottom-right at a distance of 4.5° visual angle (~251 pixels) from fixation. Place-holders stayed on the screen until the response was required at the end of the trial.

Next, two tilted grey bars (RGB value: [50,50,50]) subtending approximately 0.5° × 3.4° (~28 × 190 pixels) appeared for 250 ms inside two of these placeholders (referred to as *internal display*). The orientation of the bars was randomly determined between 0 and 180°. Participants were instructed to memorise the orientation of these two bars. There were four possible configurations of the internal display, which occurred equally often throughout the experiment: the bars could appear in the placeholders located in the (1) top-left and top-right, (2) bottom-left and bottom-right, (3) top-left and bottom-right, and (4) bottom-left and top-right. As such, at the time of the internal display, there was always one bar presented in each hemifield.

The internal display was followed by a delay of 750 ms, in which only the fixation cross and the placeholders remained on the screen. The central fixation cross then changed colour to match that of one of the placeholders for 150 ms. The colour change acted as the first cue, indicating the most likely to-be-reported item. This first cue could indicate a placeholder that previously contained an item during the internal display (i.e., retro-cue) or a placeholder in which no item had been presented yet (i.e., an item of the upcoming external display; precue). The cued location and attentional domain were pseudo-randomised such that each of the four placeholder locations and each domain was equally often indicated by the cue.

Following another delay of 750 ms, a second cue was presented by another colour change of the central fixation cross for 150 ms. This cue instructed the target item for report with a certainty of 100%, thus overruling the first cue. Accordingly, the target item was always identical to the second-cued item. In the stay condition, the colour of the second cue was identical to the colour of the first cue, therefore indicating the same item. We included 70% of stay trials in our experiment to encourage participants to use the first cue.

On 30% of the trials, the colour of the first and second cue differed, requiring a shift of attention. The shift condition was further divided into two sub-conditions: within-domain and between-domain shifts (Fig. 1B). In within-domain shift trials (external-to-external or internal-to-internal), the second cue indicated the item appearing within the opposing hemifield of the same domain as indicated by the first cue. In between-domain shift trials (internal-to-external or external-to-internal), the second cue indicated the item in the alternative domain occurring on the other hemifield. Thus, in within- and between-domain shift trials, attention always needed to be reoriented from one hemifield to the other. The second-cued location and domain were pseudo-randomised such that each of the four placeholder locations and each domain were equally often indicated.

Immediately following the second cue, a second set of randomly tilted bars appeared in the perceptual display (referred to as *external display*). The new items appeared in the yet-unoccupied placeholders for 100 ms. For example, if the bars in the internal display appeared in the top-left and bottom-right placeholders, bars in the external display occurred the bottom-left and top-right placeholders. To make the perceptual discrimination challenging, items in the external array were masked. The masking array consisted of four overlayed titled bars presented in each of the placeholders for 100 ms. At each mask location the four tilted bars differed in 45° from the neighbouring bar. The overall orientation of each mask was randomly drawn at 0 ms and 50 ms, thus creating the impression of a dynamic display. The offset of the dynamic mask was followed by a 100-ms delay, after which a visual response dial was displayed, always starting in a random position. The response dial surrounding the fixation cross was the same size as the placeholders and included markers along a circle that corresponded to the ends of a bar. The dial rotated leftwards when pressing F and rightwards when pressing J (either holding key down or pressing key repeatedly) with a speed 1/10° per millisecond. Participants reproduced the orientation of the last-cued item, with unlimited time to complete the orientation report. When the dial aligned with the orientation of the target item, participants pressed the space bar to confirm their response and continue with the task. The early onset of the external array, mask, and dial after the second cue was set to capture the costs of incomplete shifts of attention. The masking parameters were set based on piloting efforts to equate the difficulty of external and internal reports.

After a 100-ms delay in which only the fixation cross stayed on the screen, participants received feedback in the form of a number ranging from 0 to 100, with 100 indicating a perfect report and 0 indicating that the adjusted orientation was perpendicular to the angle of the target item. Feedback was presented 0.7° (~39 pixels) above the central fixation cross for 300 ms. Trials were separated by an inter-trial interval randomly drawn between 750 and 1000 ms. Between blocks participants were presented with their average reproduction accuracy in the previous block.

The experiment consisted of 640 trials divided across 16 blocks, each including 40 trials. Fig. 1C displays the proportion of conditions across the experiment. Of the total trials, 70% (448) were stay trials while the remaining 30% (192) were shift trials. Participants were equally likely to report an external or internal item in stay trials (224 each). Shift trials varied in the type of shift performed, that is, 50% (96) required a within-domain shift and the other 50% (96) a between-domain shift. Within-domain and between-domain shift trials were further split by the target domain. Ergo, the within-domain shift trials included 50% (48) external-to-external and 50% (48) internal-to-internal shifts, while the between-domain shift trials included 50% (48) internal-to-external and 50% (48) external-to-internal shifts. Each block consisted of 70% (28) stay and 30% (12) shift trials.

To become familiarised with the procedure of the experiment, participants performed four practice blocks before starting the data collection. Practice blocks had 10 trials each, consisting of 70% stay and 30% shift trials. The whole experiment lasted approximately 60 minutes.

#### 2.1.4 Behavioural analysis

Data were analysed in R Studio (Posit team, 2022). During pre-processing (similar to Gresch, Boettcher, Nobre, & van Ede, 2022; Gresch, Boettcher, van Ede, & Nobre, 2021), trials were removed when reaction times (RTs) exceeded 5000 ms. RTs refer to the interval between the appearance of the dial and the moment at which participants first started manipulating the dial. The choice of this RT measure aimed to minimise the variance related to participants’ speed and strategy when finalising and confirming their precise responses, thereby providing a useful approximation of decision time (van Ede, Niklaus, & Nobre, 2017). Next, we removed trials for which the remaining RTs were 2.5 *SD* above the individual mean across all conditions. Datasets with >10% of trials rejected during these pre-processing steps were removed from further analysis (*n* = 1). Moreover, we removed datasets with average reproduction errors (calculated by averaging the absolute difference between the angle of the target item and the reported angle) equal or higher than 45° in any of the conditions (*n* = 3). To analyse the same datasets in the behavioural and eye-tracking analysis, we removed an additional three participants due to bad eye tracking data (see ‘Eye-tracking analysis). After these exclusion steps, datasets from 25 participants remained in the main behavioural analysis, with an average of 96.88% (*SD* = 0.69) of trials retained.

The conditions of interest were *target domain* (external vs. internal), *trial type* (stay vs. shift), and *shift type* (within vs. between). When comparing more than two means, we applied a repeated-measures analysis of variance (ANOVA) and reported ${\text{η}}_{\text{G}}^{2}$ as a measure of effect size. When evaluating only two means, we applied a paired samples *t*-test and report Cohen’s *d* as a measure of effect size. For post-hoc *t*-tests, we report Bonferroni-corrected *p*-values that we denote as ‘*p*_Bonferroni_’. In the case of significant interactions, we exclusively employed post-hoc *t*-tests to identify differences between the levels of trial type or shift type within each target domain, refraining from extending these comparisons across target domains. This approach was chosen to handle potential baseline differences between external- and internal-report trials. We used the ‘ggplot2’ package (version 3.3.6; Wickham, 2009) for plotting results. The within-subject standard error of the mean was calculated from normalised data using the approach from Morey (2008).

#### 2.1.5 Eye-tracking analysis

Pre-processing and analysis procedures followed previous work from our lab (Draschkow, Nobre, & van Ede, 2022; van Ede, Chekroud, & Nobre, 2019; van Ede, Deden, & Nobre, 2021). Eye-tracking data were first converted from edf to asc format, and subsequently read into R Studio (Posit team, 2022). Eye blinks were detected and interpolated ± 100 ms around identified blinks. Data from the left and right eyes were averaged, yielding a single time course of horizontal gaze position (x-position) and vertical gaze position (y-position). Next, data were downsampled to 250 Hz (i.e., one sample every 4 ms), then epoched from 200 ms before to 1400 ms after the relative onsets of the first and second cue. To focus on biases in fixational gaze behaviour, trials were removed in which vertical or horizontal gaze positions exceeded ± 2.25° (~126 pixels) from fixation (i.e., further than half the distance to either of the four placeholders, as in Draschkow, Nobre, & van Ede, 2022; van Ede, Deden, & Nobre, 2021). We smoothed all time-course data over 15 samples (i.e., 60-ms smoothing window average) and we normalised each trial by baselining the data. For the first-cue epochs, we subtracted the mean value between −200 and 0 ms before the onset of the first cue from all subsequent time points. Similarly, for the second-cue epochs, we subtracted the mean value between −200 and 0 ms before the onset of the second cue from all following time points in the trial. Eye-tracking data from 25 participants remained in the main gaze analysis, in which an average of 93.64% (*SD* = 5.89) of trials were retained.

For simplicity, only lateral gaze biases were analysed. To compare gaze biases across trials, we first compared leftwards and rightwards gaze-position time courses within each domain relative to the first cued item. In addition, leftwards and rightwards gaze-position time courses were also compared across shift types, within each target domain and relative to the second cue. Time-course data were plotted in a 1600-ms window beginning 200 ms prior to the relative cue (i.e., first or second cue). To increase sensitivity and interpretability, we calculated the mean of the lateral gaze bias according to the location of the cued item (i.e., towardness; as in Draschkow, Nobre, & van Ede, 2022; van Ede, Chekroud, & Nobre, 2019; van Ede, Deden, & Nobre, 2021). Towardness for each time step was given by the trial-average horizontal position in right-item trials minus the trial-average horizontal position in left-item trials (where position values left of fixation were negative) divided by two. A positive towardness indicated a gaze bias in the direction of the cued item. We used the towardness variable to determine the significance of gaze biases (compared to zero), as well as to test each time point of gaze biases for differences between conditions. We ran cluster-based permutation testing with the ‘permuco’ package (Frossard & Renaud, 2021), using ‘cluster-mass’ permutation tests with 5000 permutations, which controls for multiple comparisons across time points while retaining high sensitivity.

Moreover, we computed cross-correlations to test for potential latency differences between towardness time courses. Calculating cross-correlations involves shifting one signal relative to the other and computing the correlation coefficient of the two signals at each shift (i. e., lag). The resulting cross-correlation function indicates how well the two signals match at each shift, that is, a high cross-correlation value at a specific time lag indicates a strong relationship between the two signals at that time lag. To accurately determine the lag at which the cross-correlation reached its maximum, we employed two methods. Firstly, we identified individual peaks in the cross-correlation. Additionally, we utilised a jack-knife approach to enhance our analysis. For the jack-knife approach, we computed cross-correlation averages by excluding each of the *n* data sets once, allowing us to obtain *n* leave-one-out grand averages. Next, we identified the lags at which the cross-correlation coefficient reached its maximum. When possible, jack-knifed standard errors, *t*-values, and *p*-values were adjusted according to Miller, Patterson, and Ulrich (1998).

For visualisation purposes, we constructed heat maps of gaze positions between 400 and 1400 ms following the first cue. For these analyses, we did not remove trials with gaze values exceeding more than half the distance of the placeholder centres. Two-dimensional kernel density estimations were obtained at a 125 × 125-pixel spacing on a 500 × 500-pixel square grid separately for cues indicating right and left placeholders. Lastly, we subtracted density maps of left- from right-cued items which allowed us to illustrate the magnitude of the observed gaze bias.

### 2.2 Results

#### 2.2.1 Shifting attention is associated with performance costs

As we have developed a task for quantifying the costs of unfolding shifts of attention, our initial analyses focused on confirming the success of the experimental design in this regard. Specifically, we tested whether participants shifted their attention according to the initial cue before reorienting according to the second cue. Since the second cue was always 100% instructive for the later report, it was necessary to confirm that participants also engaged with the first cue. If participants made use of the highly predictable first cue, performance should be better in stay trials wherein the cued information was repeated across the first and second cue and therefore required no subsequent shift of attention.

**Fig. 2:**
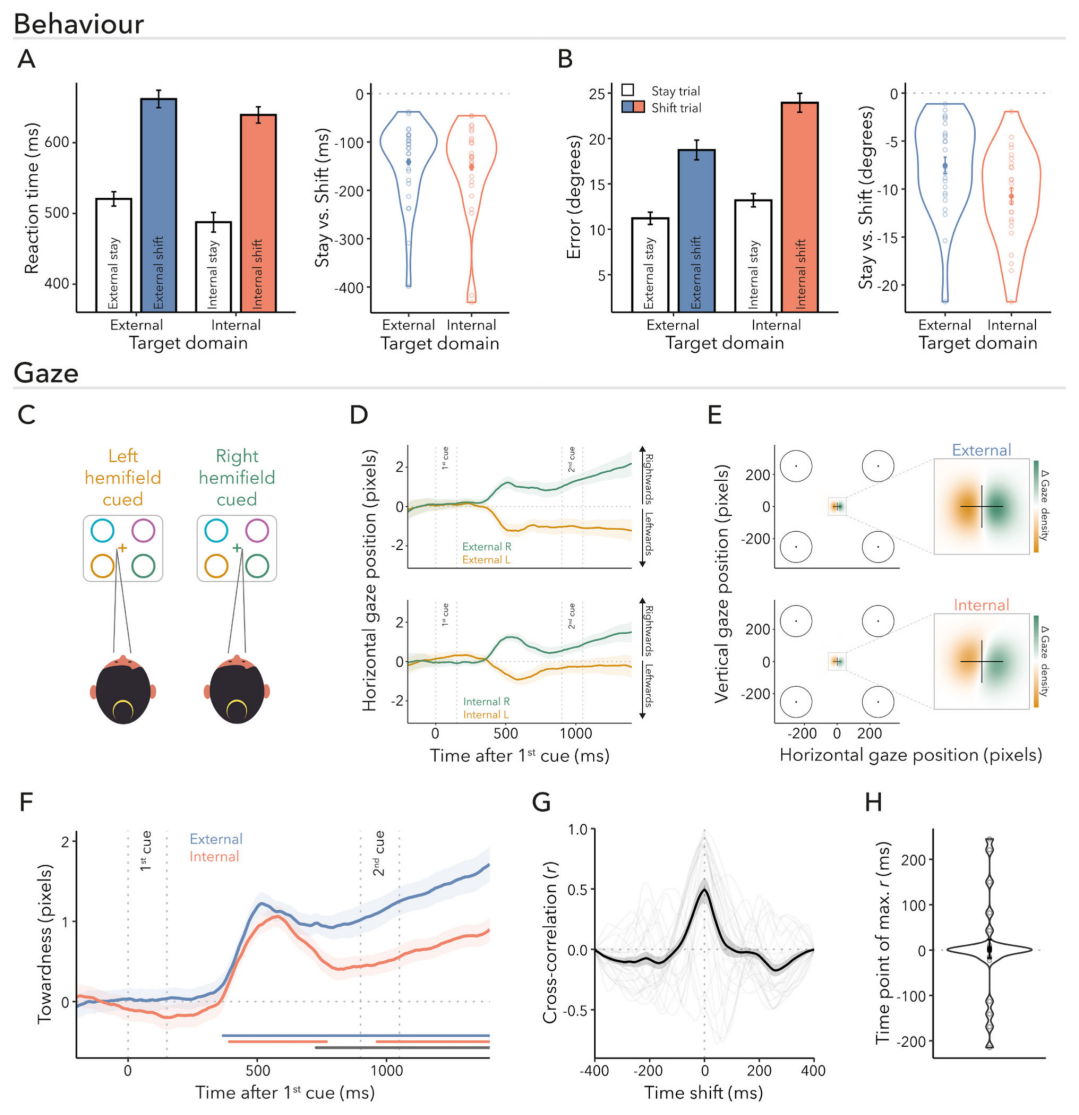
Behavioural and gaze effects of orienting attention. (A) Reaction times were slower for shift than for stay trials and for external- than for internal-target trials. (B) Reproduction errors were higher for shift than for stay trials, whereby the effect was larger for internal- than for external-target trials. (C) Schematic depicting predicted gaze biases following left and right cues. (D) Average gaze position (in the horizontal plane) after the onset of an external (top panel) and internal (bottom panel) first cue as a function of time and the location of the first-cued item. (E) Difference in gaze density (Δ) following cues for right minus left items (400–1400 ms post first cue) for external (top panel) and internal (bottom panel) first-cue trials. (F) Gaze bias expressed as ‘towardness’ for external and internal first cues. Horizontal lines denote significant clusters (grey line denotes external vs. internal). (G) Cross-correlation coefficients of the external and internal towardness time courses. (H) Average lag at which the correlation-coefficient reached its maximum. Error bars and shaded areas indicate ±1 SE. Dots represent individual participants.

We evaluated RTs (i.e., interval between dial onset and initial dialling response) and compared stay versus shift trials for both external- and internal-target reports (Fig. 2A). We observed a significant main effect of trial type (*F*_(1,24)_ = 66.840, *p* < 0.001, ${\text{η}}_{\text{G}}^{2}=0.130$). Trials that required participants to shift their attention to a different item based on the second cue resulted in slower RTs compared to trials where the second cue was redundant, allowing participants to maintain their attention on the same item. This suggests that participants relied on the information provided by the first cue. We also found a significant main effect of target domain, indicating faster reports for internal compared to external targets (*F*(_1,24)_ = 8.104, *p* = 0.009, ${\text{η}}_{\text{G}}^{2}=0.005$). The interaction between trial type and target domain was not significant (*F*_(1,24)_ = 1.516, *p* = 0.230, ${\text{η}}_{\text{G}}^{2}<0.001$). We observed the same statistical pattern when analysing response-completion times, that is, response times based on the interval between the appearance of the dial and participants’ confirmation of their responses by pressing space bar (Supplementary Fig. 2B).

We also considered the influence of trial type and target domain on reproduction errors (Fig. 2B). Errors were systematically larger when participants were required to shift attention (*F*_(1,24)_ = 147.301, *p* < 0.001, ${\text{η}}_{\text{G}}^{2}=0.332$), again consistent with an initial use of the first cue. This was true for all participants in our experiment (Fig. 2B, right panel), as it was also the case for RTs (Fig. 2A, right panel). There was also a significant effect of target domain (*F*_(1,24)_ = 8.590, *p* = 0.007, ${\text{η}}_{\text{G}}^{2}=0.071$), whereby internal items were reported with higher errors than external items. In contrast to RTs, trial type and target domain interacted for errors (*F*_(1,24)_ = 6.997, *p* = 0.014, ${\text{η}}_{\text{G}}^{2}=0.015$). Pairwise comparisons revealed that the difference between stay and shift trials was more pronounced when reporting the orientation of internal targets as compared to external targets (*t*_(24)_ = 2.645, *p*_Bonferroni_ = 0.043, *d* = 0.529). However, shift costs for errors were significant for both external-target (*t*_(24)_ = 7.742, *p*_Bonferroni_ < 0.001, *d* = 1.548) and internal-target trials (*t*_(24)_ = 11.169, *p*_Bonferroni_ < 0.001, *d* = 2.234).

#### 2.2.2 Fixational gaze behaviour tracks orienting of attention in perception and working memory

To obtain further verification that participants utilised the first cue, we next focussed our analysis on fixational gaze behaviour relative to the first-cue onset (Fig. 2C). Fig. 2D depicts the average horizontal gaze position following external and internal cues (top and bottom panels, respectively) for trials with first-cued items to the left (orange) and right (green) of fixation. We observed noticeable gaze shifts towards the direction of the cued location, that is, when the first-cued item occupied a left (right) position on the screen, gaze became biased to the left (right). Density maps of gaze positions contrasting right- and left-cued trials show that this bias was primarily composed of small displacements towards the location of the cued content (Fig. 2E; top panel: external cue, bottom panel: internal cue), consistent with previously demonstrated biases in fixational gaze behaviour (Draschkow et al., 2022; Engbert & Kliegl, 2003; Hafed et al., 2011; van Ede, Chekroud, & Nobre, 2019; van Ede, Deden, & Nobre, 2021).

To quantify these lateral gaze biases, we combined the right- and left-cued gaze time courses into a single ‘towardness’ metric for each domain (Fig. 2F). Cluster-based permutation analyses demonstrated a large significant cluster for external (blue horizontal line; *p* < 0.001) and two significant clusters for internal first-cue trials (red horizontal lines; first cluster: *p* = 0.006; second cluster: *p* = 0.020), indicating that both domains biased gaze towards the cued location. This result complements our behavioural findings and can be taken as additional evidence that participants utilised the first cue when performing the task. The towardness metric additionally allowed us to compare the time course of attentional shifts between the two domains directly. Interestingly, when comparing gaze biases between the external and internal domains, we observed a significant cluster only after the initial peak that was common across domains (Fig. 2F; grey horizontal line; *p* = 0.015), suggesting that external attention evoked a more sustained bias than internal attention. The towardness time courses of all conditions are illustrated in Supplementary Fig. 3A and B.

The onset timing for external and internal gaze biases appeared similar by visual inspection. Since statements regarding the exact onset of an effect are not supported by permutation tests (Sassenhagen & Draschkow, 2019), we calculated cross-correlations to characterise the temporal relationship between external- and internal-orienting mechanisms (Fig. 2G). A high cross-correlation coefficient indicates a strong similarity between two signals at a particular time lag, while a low co-efficient indicates dissimilarity. In other words, it determines how much one signal lags or leads another signal at various points in time. Fig. 2H shows the time lags of the maximal cross-correlations when comparing first-cue trials between domains. Notably, there was no temporal shift in gaze compared to lag zero (*t*_(24)_ = 0.140, *p* = 0.890, *d* = 0.028), suggesting that there was likely no latency difference in gaze when orienting attention externally versus internally. To quantify the observed cross-correlations more formally, we also applied a jack-knife approach which yielded the same result (*M* = 0; *SE, t*-value, and *p*-value were not determinable due to lack of variance).

Our findings indicate that the latencies of gaze biases were comparable for external and internal first-cued items. However, we observed that external compared to internal orienting elicited a more prolonged and sustained gaze bias. Most important for the current purpose is that, collectively, these results suggest that participants relied on the first cue to successfully complete the task.

#### 2.2.3 Higher performance costs when shifting attention between compared to within domains

**Fig. 3:**
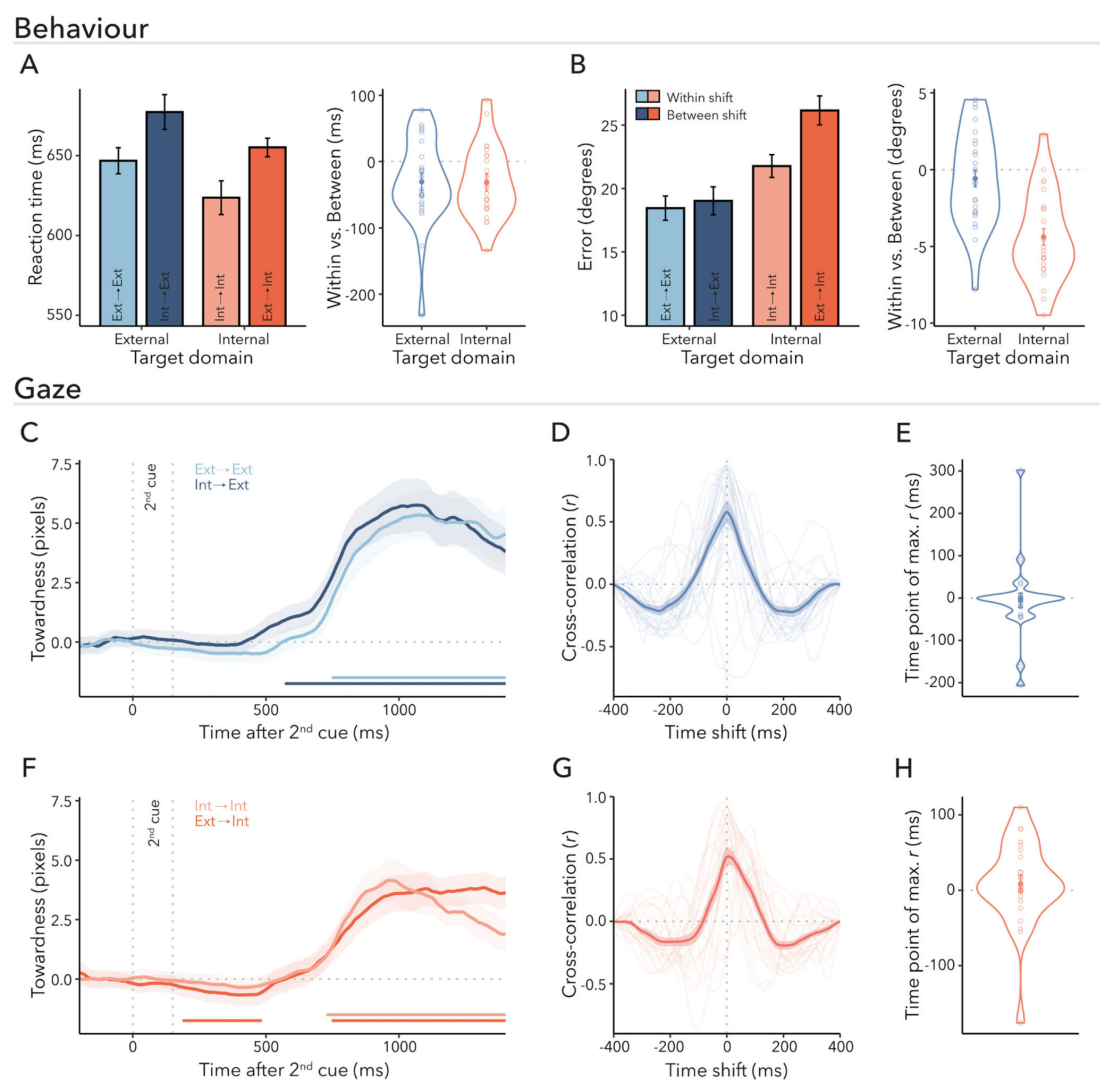
Between-domain shift costs for behaviour but not for gaze biases. (A) Reaction times were slower when shifting between as compared to within domains and for external- as compared to internal-target reports. (B) The effect of shift type on errors depended on the level of target domain. There was only a between-domain shift cost when reporting internal but not external targets. (C) Gaze bias expressed as ‘towardness’ for external within- and between-domain shift trials. Horizontal lines denote significant clusters. (D) Cross-correlation coefficients for the within- and between-shift time courses, evaluated for external-target trials. (E) Average time point (i.e., lag) at which the correlation-coefficient reached its maximum for external-target trials. (F) Same as (C) but for internal-target trials. (G) Same as (D) but for internal-target trials. (H) Same as (E) but for internal-target trials. Error bars and shaded areas indicate ±1 SE. Dots represent individual participants. Ext → Ext = external-to-external, Int → Int = internal-to-internal, Int → Ext = internal-to-external, Ext → Int = external-to-internal.

Having established that participants utilised the first cue, we next turned to the behavioural dynamics of shifting attention within versus between domains (Fig. 3A and B). Thus, the following analyses focuses on the shift trials in which participants were required to reorient attention within or between domains. If shifting attention between was more costly than shifting within domains, performance should be slower and worse for internal-to-external than external-to-external and for external-to-internal than internal-to-internal trials. We tested this using a repeated-measures ANOVA with shift type (within vs. between) and target domain (external vs. internal) as factors. RTs were slower for between- compared to within-domain shifts (Fig. 3A; *F*_(1,24)_ = 16.388, *p* < 0.001, ${\text{η}}_{\text{G}}^{2}=0.005$) and for reporting external compared to internal targets (*F*_(1,24)_ = 4.947, *p* = 0.036, ${\text{η}}_{\text{G}}^{2}=0.003$). The interaction between target domain and shift type was not significant (*F*_(1,24)_ = 0.003, *p* = 0.957, ${\text{η}}_{\text{G}}^{2}<0.001$). The same statistical pattern was observed when analysing response-completion times instead of RTs (Supplementary Fig. 2D).

As depicted in Fig. 3B, reproduction errors were higher when participants had to shift between domains (*F*_(1,24)_ = 29.859, *p* < 0.001, ${\text{η}}_{\text{G}}^{2}=0.023$). Again, we found a main effect of target domain (*F*_(1,24)_ = 9.660, *p* = 0.005, ${\text{η}}_{\text{G}}^{2}=0.093$), with errors being higher for internal than external targets. Unlike for RTs, for errors there was also a significant interaction between target domain and shift type (*F*_(1,24)_ = 25.040, *p* < 0.001, ${\text{η}}_{\text{G}}^{2}=0.014$), suggesting that the shift type had a different effect on errors depending on the target domain. The between-domain shift cost was higher for reporting internal targets compared to external targets, that is, external-to-internal versus internal-to-internal shifts were less accurate than internal-to-external versus external-to-external shifts (*t*_(24)_ = 5.004, *p*_Bonferroni_ < 0.001, *d* = 1.001). Additional pairwise post-hoc comparisons revealed that there was a significant difference between internal-to-internal and external-to-internal shifts (*t*_(24)_ = 7.740, *p*_Bonferroni_ < 0.001, *d* = 1.548). However, the difference between external-to-external and internal-to-external shifts was not significant (*t*_(24)_ = 0.936, *p*_Bonferroni_ = 1.000, *d* = 0.187).

In sum, we found evidence for higher performance costs in RTs when shifting attention between as opposed to within domains. For accuracy, there was only a cost of shifting between domains when the final target was selected from the internal attentional domain. A consistent result pattern emerged when utilising a percentage-change measure to normalise the between-domain shift-cost effect across each target domain (i.e., removing any influence of between-domain baseline performance levels; Supplementary Fig. 4).

#### 2.2.4 No evidence for differences in gaze biases when shifting within versus between domains

After having observed higher performance costs for between-domain shifts compared to within-domain shifts, we asked whether this cost was also reflected in the gaze biases. Specifically, we aimed to investigate whether there was evidence supporting the notion that shifting attention between domains would result in delayed gaze biases compared to shifting within domains. This effect could potentially indicate the presence of an additional obstacle or boundary to overcome during the attentional shifting process, aligning with the observed impact on RTs. We again focused on shift trials.

First, we computed the aggregated measure of towardness and analysed the time of within- and between-domain shifts following the second-cue onset for external-target (Fig. 3C) and internal-target trials (Fig. 3F). After attention was prompted to shift between contents, late clusters emerged for all the four shift types (external-to-external: *p* < 0.001; internal-to-internal: *p* < 0.001; internal-to-external: *p* < 0.001; external-to-internal: *p* < 0.001; light blue, light red, dark blue, dark red horizontal lines, respectively), indicating the second cue biased gaze towards the direction of the target item. In addition, we observed a gaze bias in the direction of the first-cued item for external-to-internal trials (dark red horizontal line; *p* = 0.047). No clusters were found when comparing within- versus between-domain shift trials separately within either domain.

Besides comparing the magnitude of gaze biases, we were particularly interested in the latencies of these biases following external and internal within- and between-domain shifts. Fig. 3D and G illustrate the cross-correlations when examining within-domain and between-domain shifts for each respective domain. There was no evidence for a temporal shift in gaze compared to lag zero for either external- (Fig. 3E; *t*_(24)_ = −0.308, *p* = 0.761; *d* = 0.062) or internal-target trials (Fig. 3H; *t*_(24)_ = 0.756, *p* = 0.457; *d* = 0.151). These results were replicated using a jack-knife approach (external-target trial: *M* = 0, *SE, t*-value, and *p*-value were not determinable due to lack of variance; internal-target trial: *M* = 6.120, *SE* = 8.465, *t*_(24)_ = 0.723, *p* = 0.477). Therefore, our findings did not reveal a significant delay in gaze biases when shifting attention between domains relative to within domains, despite the observed slowing of behavioural reporting following between–domain shifts.

### 2.3 Summary

In sum, Experiment 1 led to several key findings. First, our task design was effective at eliciting attention shifts to external and internal contents as evidenced by behavioural and gaze-bias findings. Second, shifting attention between domains was associated with a higher behavioural cost than shifting attention within domains. Third, the between-domain shift cost was asymmetric for reproduction errors; within- versus between-domain shifts differed for internal- but not for external-target reports. And lastly, in contrast to performance measures, gaze position measures were insensitive to whether shifts occurred within or between domains. The divergence between behavioural results and the gaze bias suggests that the cost associated with shifting attention between domains is not primarily driven by the spatial orienting functions, which the gaze bias likely reflects.

## 3 Experiment 2

The second, follow-up experiment was designed to uncover putative differences in the temporal dynamics of shifting attention within versus between domains by systematically varying the duration between the second cue and the external display. This allowed us to disambiguate potential explanations of the observed shift-cost effects. For example, specific patterns of performance costs could be accounted for by an extended period being necessary to complete attentional orienting, selection, prioritisation, and/or response gating, when shifting between domains, or from one particular domain to another. If this were the case, providing additional time to shift attention after the second cue should mitigate any increased or asymmetric costs. Furthermore, the presence of the asymmetric shift cost in reproduction errors might have been contingent on participants having minimal time to recall the internal target after the second cue. A similar memory-retrieval account of between-domain shifts has been proposed by Dark (1990).

In addition to manipulating the intervals after the second cue, Experiment 2 also encouraged the reliance on cues in a different and more economical manner. Rather than including a large proportion of stay trials, the design included one third of trials in which the external display appeared after the first cue, prompting a response to the initially cued item. The different approach was also aimed at testing the robustness of the pattern of results from Experiment 1.

### 3.1 Methods

#### 3.1.1 Participants

Again, all experimental procedures were approved by the Central University Research Ethics Committee of the University of Oxford. Participant numbers were derived from applying the same power analysis we used previously to the full data set of Experiment 1 (*n* = 25). The power analysis indicated that a sample size of *n* = 25 would yield power exceeding the 80% threshold even if the RT effect size was 30% smaller (Supplementary Fig. 1B).

We collected data from 26 participants. One participant was excluded following our a-priori behavioural trial-removal procedure (see ‘Behavioural analysis’). All remaining 25 participants (age range: 19 to 39; mean age: 26.92; gender: 16 female, 7 male, 2 non-binary; handedness: 23 right-handed, 2 left-handed) had normal or corrected-to-normal vision. Individuals provided written informed consent before participating in the study and were paid £15 per hour.

#### 3.1.2 Apparatus and data acquisition

Participants sat in front of a monitor (23.6-in. DELL E2417H; resolution 1920 × 1080 pixels; refresh rate 60 Hz; screen width 53 cm) at a viewing distance of 80 cm. The experimental script was generated in Python using the ‘PsychoPy’ package (version 2021.2.3; Peirce et al., 2019).

#### 3.1.3 Task and procedure

**Fig. 4:**
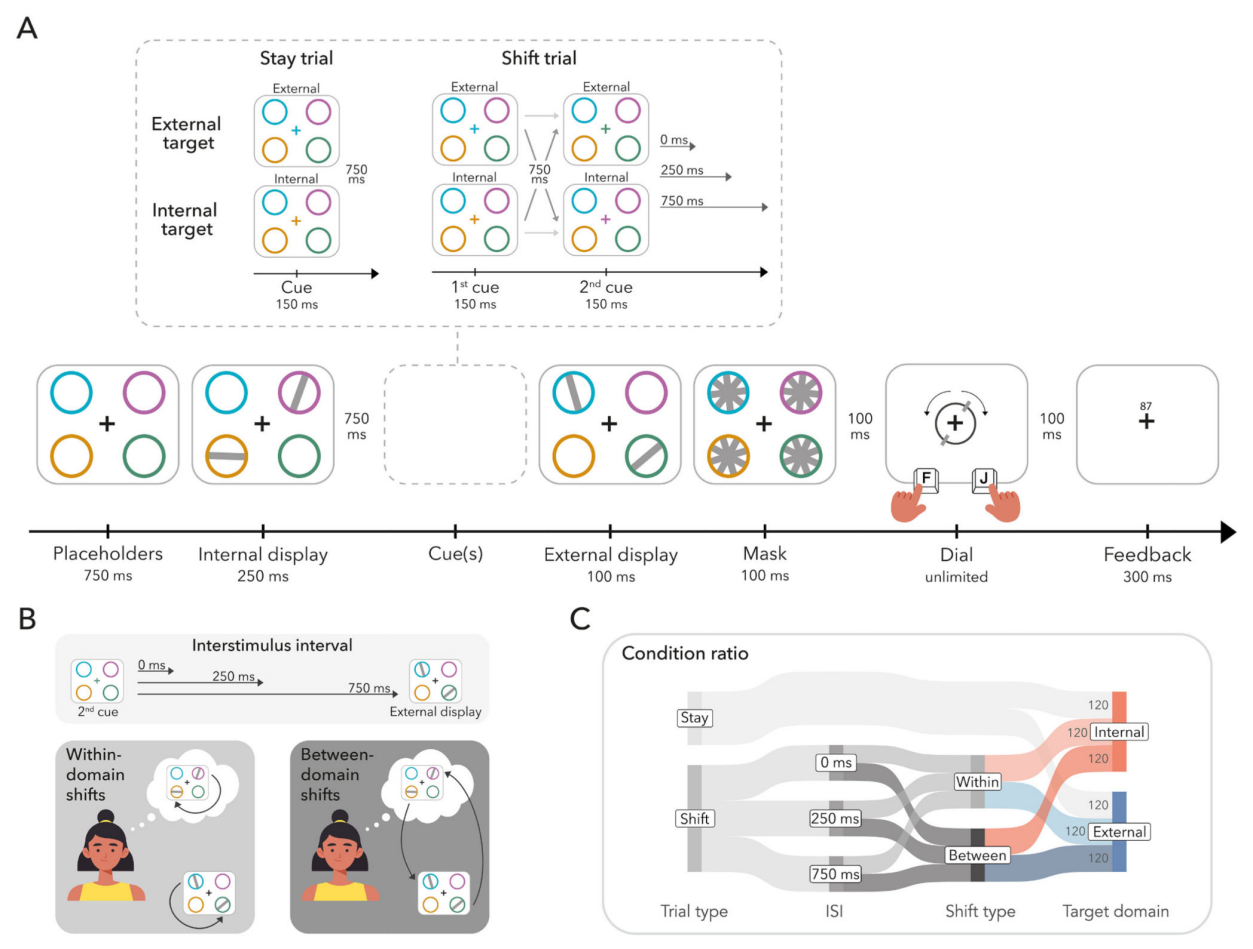
Task schematic and condition details of Experiment 2. (A) Illustration of stay and shift trials. In stay trials, only a single cue was presented. In shift trials, the presentation of the second cue was followed by an interstimulus interval of either 0 ms, 250 ms, or 750 ms. Light-grey arrows depict within-domain shifts and dark-grey arrows represent between-domain shifts. (B) In within-domain shift trials, participants either had to shift attention external-to-external or internal-to-internal. In between-domain shift trials, participants either had to shift attention external-to-internal or internal-to-external. Additionally, we manipulated the interstimulus interval between the second cue and the external display in shift trials. (C) Ratio of conditions and number of trials across the experiment.

Experiment 2 closely adhered the procedures from Experiment 1, with three notable deviations (Fig. 4A and B). Firstly, to delve into the temporal dynamics of the between-domain shift cost, the inter-stimulus-interval (ISI) between the second cue and the external display was manipulated in shift trials. The ISI could either be 0 ms (same as Experiment 1), 250 ms, or 750 ms. Secondly, the stay condition contained only a single cue (i.e., single-cue condition). The cue in stay trials appeared at the time of the first cue in shift trials and was then followed by an ISI of 750 ms before the onset of the external display. Participants were instructed to always report the last-cued item. That is, in shift trials, participants reported the second-cued item (same as Experiment 1), however, in stay trials, participants reported the item indicated by the single cue. Lastly, the proportion of stay and shift trials differed in comparison to Experiment 1 (Fig. 4C).

The experiment consisted of 720 trials divided across 20 blocks, each including 36 trials. Of the total trials, 13 (240) were stay trials. The remaining 23 (480) were shift trials. Participants were equally likely to report an external or internal item in both stay trials (120 each) and shift trials (240 each). Within shift trials, within- and between-domain shifts were equiprobable (240 trials each). Thus, the four shift conditions each included 120 trials, which were further divided into 40 trials of each equally possible ISIs. Each block consisted of 13 (12) stay and 23 (24) shift trials.

#### 3.1.4 Behavioural analysis

Data were first pre-processed according to the pipeline matching that of Experiment 1. During pre-processing, trials were removed when RTs exceeded 5000 ms. Next, we removed trials for which the remaining RTs were 2.5 *SD* above the individual mean across all conditions. None of the participant was removed for loss of >10% of trials. One dataset was removed due to average reproduction errors exceeding 45° in a subset of conditions. After these exclusion steps, datasets from 25 participants remained in the main behavioural analysis, with an average of 97.01% (*SD* = 0.55) of trials retained. The conditions of interest were *target domain* (external vs. internal), and *shift type* (within vs. between) and *ISI* (0 ms vs. 250 ms vs. 750 ms). As in Experiment 1, the dependent variables of interest were RTs and reproduction errors.

While trial numbers were too low in Experiment 1, Experiment 2 allowed us to assess the proportion of trials in which participants incorrectly reported the angle of the first-cued item (i.e., swaps, non-target reports) as well as the proportion of trials in which random guessing occurred. For each participant and condition, we fitted a probabilistic mixture model and estimated the relative proportions of the non-target (i.e., first-cued item) distribution as well as the guessing distribution (Bays, Catalao, & Husain, 2009). The mixture modelling analysis was performed using the ‘mixtur’ package (version 1.2.1; Grange & Moore, 2022).

When comparing more than two means, we applied a repeated-measures analysis of variance (ANOVA) and reported ${\text{η}}_{\text{G}}^{2}$ as a measure of effect size. When evaluating only two means, we applied a paired samples *t*-test and report Cohen’s *d* as a measure of effect size. For post-hoc *t*-tests, we report Bonferroni-corrected *p*-values that we denote as ‘*p*_Bonferroni_’.

### 3.2 Results

#### 3.2.1 Between-domain shift costs are not modulated across time

**Fig. 5:**
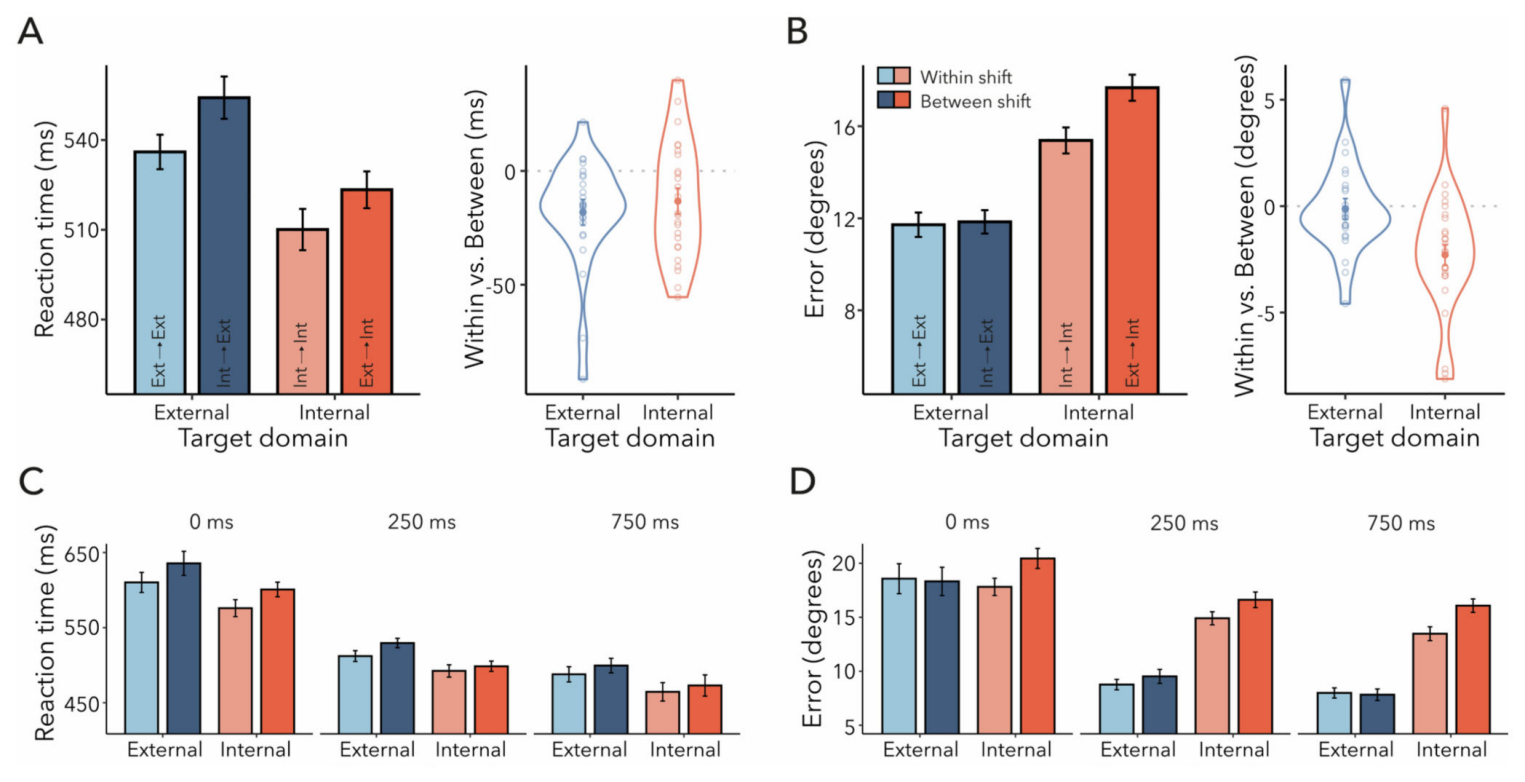
No modulation of between-domain shift costs across time. (A) Reaction times were slower when shifting between as compared to within domains and for external as compared to internal reports. (B) The effect of shift type on reproduction errors depended on the level of target domain. There was only a between-domain shift cost when reporting internal but not external targets. (C) The shift-cost effect in reaction times was not modulated by the ISI condition. (D) The shift-cost effect in reproduction errors was not modulated by the ISI condition. Error bars indicate ±1 SE. Dots represent individual participants. Ext → Ext = external-to-external, Int → Int = internal-to-internal, Int → Ext = internal-to-external, Ext → Int = external-to-internal.

The following analyses focused on the shift trials in which participants reoriented attention within or between domains. We used a repeated-measures ANOVA with shift type (within vs. between), target domain (external vs. internal), and ISI (0 ms vs. 250 ms vs. 750 ms) as factors. As in Experiment 1, RTs were slower for between- compared to within-domain shifts (Fig. 5A; *F*_(1,24)_ = 29.124, *p* < 0.001, ${\text{η}}_{\text{G}}^{2}=0.004$) and for reporting external compared to internal targets (*F*_(1,24)_ = 7.635, *p* = 0.011, ${\text{η}}_{\text{G}}^{2}=0.012$). Moreover, there was a significant effect of ISI (Fig. 5C; *F*_(2,48)_ = 59.565, *p* < 0.001, ${\text{η}}_{\text{G}}^{2}=0.150$). Post-hoc comparisons revealed that RTs became faster with increasing ISI Participants responded faster in trials with a 250-ms ISI as compared to a 0-ms ISI (*t*_(24)_ = 9.891, *p*_Bonferroni_ < 0.001, *d* = 1.978), and faster still in trials with a 750-ms ISI as compared to a 250-ms ISI *t*_(24)_ = 3.244, *p*_Bonferroni_ = 0.010, *d* = 0.649). Whether shift type interacted with ISI was a central question in Experiment 2. No interactions involving ISI were observed and no other interaction effect reached significance (see Supplementary Table 1A for the full set of interferential statistics).

Reproduction errors also showed a similar pattern as in Experiment 1. Participants made more errors when they shifted attention between compared to within domains (Fig. 5B; *F*_(1,24)_ = 11.019, *p* = 0.003, ${\text{η}}_{\text{G}}^{2}=0.015$). Again, we found a main effect of target domain (*F*_(1,24)_ = 34.158, *p* < 0.001, ${\text{η}}_{\text{G}}^{2}=0.186$), with errors being higher for internal than external targets. Additionally, we observed a main effect of ISI (Fig. 5D; *F*_(2,48)_ = 82.596, *p* < 0.001, ${\text{η}}_{\text{G}}^{2}=0.306$). Errors became smaller with increasing ISI. Errors were smaller at an ISI of 750 ms than at an ISI of 250 ms (*t*_(24)_ = 3.201, *p*_Bonferroni_ = 0.011 *d* = 0.640), which, in turn, were smaller than at an ISI of 0 ms (*t*_(24)_ = 9.162, *p*_Bonferroni_ < 0.001, *d* = 1.832).

As in Experiment 1, there was also a significant interaction between target domain and shift type (*F*_(1,24)_ = 10.282, *p* = 0.004, ${\text{η}}_{\text{G}}^{2}=0.012$). The between-domain shift cost was higher for reporting internal targets compared to external targets, that is, external-to-internal versus internal-to-internal shifts were less accurate compared to internal-to-external versus external-to-external shifts (*t*_(24)_ = 3.162, *p*_Bonferroni_ = 0.013, *d* = 0.632). Additional pairwise post-hoc comparisons revealed a significant difference between internal-to-internal and external-to-internal shifts (*t*_(24)_ = 4.010, *p*_Bonferroni_ = 0.002, *d* = 0.802). However, the difference between external-to-external and internal-to-external shifts was not significant (*t*_(24)_ = 0.303, *p*_Bonferroni_ = 1.000, *d* = 0.061). The pattern of effects, therefore, matched that in Experiment 1.

The critical interaction between shift type and ISI did not reach significance and there was also no three-way interaction (see Supplementary Table 1B for the full set of interferential statistics). We did however observe an interaction between ISI and target domain (*F*_(2,48)_ = 12.726, *p* < 0.001, ${\text{η}}_{\text{G}}^{2}=0.077$). Errors were significantly higher for internal as compared to external targets at the 250-ms (*t*_(24)_ = 7.943, *p*_Bonferroni_ < 0.001, *d* = 1.589) and 750-ms ISI (*t*_(24)_ = 11.566, *p*_Bonferroni_ < 0.001, *d* = 2.313), but there was no difference between target domains at the 0-ms ISI (*t*_(24)_ = 0.385, *p*_Bonferroni_ = 1.000, *d* = 0.077).

Taken together, we replicated the pattern of findings from Experiment 1. Additionally, we found no evidence to suggest that the between-domain shift costs were modulated by the ISI.

#### 3.2.2 No evidence for higher misreporting errors when shifting attention between domains

The mixture-modelling approach provided an additional perspective on the factors contributing to performance effects. According to mixture modelling, errors in a continuous-report task can arise due to two potential sources (Bays et al., 2009). Participants could accidentally swap the target with one of the other items or randomly guess. Hence, one plausible explanation for the asymmetric between-domain cost in reproduction errors could be that participants committed more swap errors when shifting from the external to the internal domain. Since the shift cost in reproduction errors was not modulated by the ISI and to improve the fit of our model, we trained a probabilistic mixture model for each combination of target domain and shift type (i.e., without further splitting the data by ISI).

**Fig. 6:**
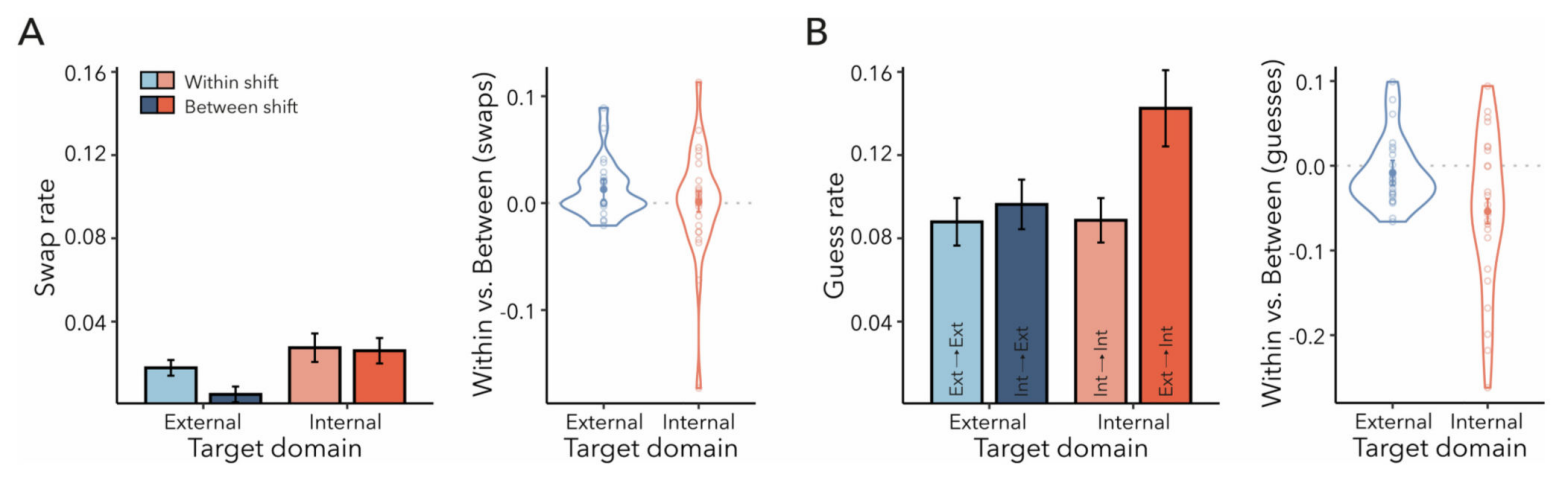
Swap and guess rates. (A) Proportion of swaps as a function of target domain and shift type. Swaps with the first-cued item were more likely in internal- as compared to external-target trials. (B) Proportion of guesses as a function of target domain and shift type. Between- as opposed to within-domain shifts significantly increased the probability of guesses. Moreover, the effect of shift type was modulated by the level of target domain. Error bars indicate ±1 SE. Dots represent individual participants. Ext → Ext = external-to-external, Int → Int = internal-to-internal, Int → Ext = internal-to-external, Ext → Int = external-to-internal.

Fig. 6A depicts the rate of swap errors. The proportion of swaps was very low across all conditions (*M* = 0.019; *SE* = 0.005) and could not account for the larger reproduction-error costs when shifting between as compared to within domains in internal-target trials. Neither the effect of shift type (*F*_(1,24)_ = 2.182, *p* = 0.153, ${\text{η}}_{\text{G}}^{2}=0.016$) nor the interaction between shift type and target domain (*F*_(1,24)_ = 0.699, *p* = 0.411, ${\text{η}}_{\text{G}}^{2}=0.010$) reached significance. We did, however, observe more swaps for internal- compared to external-target trials (*F*_(1,24)_ = 15.408, *p* < 0.001, ${\text{η}}_{\text{G}}^{2}=0.072$).

The proportion of guesses was approximately five-times larger than the proportion of swaps (*M* = 0.104, *SD* = 0.013) and followed the asymmetric performance costs (Fig. 6B). A significant interaction between shift type and target domain was observed (*F*_(1,24)_ = 4.795, *p* = 0.038, ${\text{η}}_{\text{G}}^{2}=0.020$). Post-hoc pairwise comparisons showed significantly more guesses for external-to-internal than internal-to-internal shifts (*t*_(24)_ = 2.947, *p*_Bonferroni_ = 0.014, *d* = 0.589). However, no difference in guesses occurred between internal-to-external and external-to-external shifts (*t*_(24)_ = 1.017, *p*_Bonferroni_ = 0.638, *d* = 0.203). A main effect of shift type showed a larger probability of guesses in between- than within-domain shift trials (*F*_(1,24)_ = 10.425, *p* = 0.004, ${\text{η}}_{\text{G}}^{2}=0.037$). The target domain did not influence the guessing rate significantly (*F*_(1,24)_ = 1.612, *p* = 0.216, ${\text{η}}_{\text{G}}^{2}=0.021$).

In sum, the mixture-model analysis suggested that misreporting the first-cued item did not explain the asymmetric between-domain shift cost observed in reproduction errors. Instead, a greater proportion of guessing occurred when shifting from the external to internal domain compared to shifting within the internal domain.

### 3.3 Summary

In Experiment 2, we successfully replicated the results obtained in Experiment 1. Participants consistently exhibited slower response times when shifting their attention between domains compared to shifting within the same domain. Furthermore, we again observed an asymmetric shift cost in reproduction errors, indicating that external-to-internal versus internal-to-internal shifts incurred higher costs than internal-to-external versus external-to-external shifts. Given the variations in task design and condition ratio between Experiment 1 and Experiment 2, our findings demonstrate good generalisability of the within- versus between-domain shift effects. In Experiment 2 it also became evident that the observed effects remained consistent regardless of the allocated time for attentional shifts. This implies that the attentional system did not fully recover from the consequences of shifting between domains within the tested ISIs. Moreover, the asymmetric between-domain shift costs in reproduction errors could not be attributed to swaps with the initially cued item. Taken together, the findings from Experiment 2 suggest the possible existence of a distinct, additional control function engaged during shifts of attention between domains.

## 4 General discussion

The aim of the present studies was to initiate a new line of research investigating a fundamental yet less explored aspect of everyday cognition, that is, shifting attention between external sensory signals and internal contents in working memory. In two experiments, participants were asked to reorient their attention to equivalent stimulus attributes in perception and working memory to achieve a common task goal, thereby echoing numerous real-life situations. Take, for example, the task of building a Lego set, in which our attention constantly shifts between the various sensory contents (i.e., the instructions, the pile of Lego pieces, and the partially assembled model) and mental representations (i.e., visualising the required pieces, how they fit together, and their placement within the model). Thus, our task both complements and differs from previous studies, which have focused on the costs associated with shifting between trials relying on external versus internal information (Calzolari et al., 2022; Carlson et al., 1993; Dark, 1990; Gilbert et al., 2005; Hautekiet et al., 2023; Verschooren et al., 2020; Verschooren, Liefooghe, et al., 2019; Weber, Burt, & Noll, 1986) and utilised different stimulus material for the external and internal domain (Verschooren et al., 2021; Verschooren, Schindler, et al., 2019).

Our newly developed tasks proved effective in assessing the performative consequences of shifting attention within versus between perceptual and mnemonic contents. In Experiment 1, we observed significantly higher costs in both RTs and errors for shift as compared to stay trials and additionally revealed that the costs associated with shifting attention were notably higher when reorienting between different domains as opposed to within the same domain. Experiment 2 replicated these findings and demonstrated that participants were not able to recover from the between-domain shift costs even when more time was provided to shift attention. The continuous proxy of spatial orienting in the form of small fixational gaze biases confirmed participants utilised both cues during the task and further uncovered interesting dissociations between the spatial orienting functions tracked by the oculomotor biases and the ultimate consequences to task performance as reflected in the behavioural performance measures.

### 4.1 Successful orienting and reorienting of external and internal attention

Our results yielded compelling evidence that participants utilised the cues to guide their attention. In Experiment 1, RTs were slower and reproduction errors greater when required to reorient attention to a different item. These findings align with previous research showing behavioural costs when being invalidly cued (Griffin & Nobre, 2003; Posner, 1980). Hence, the highly predictable first cue incentivised participants to focus their attention on the cued location, enabling us to detect attentional costs ‘mid-flight’ when the dial appeared. Our eye-tracking measures provided convergent evidence that participants utilised the first cue. Fixational eye movements offer a continuous proxy for the dynamics of spatial shifts of attention in the external and internal domains (Draschkow et al., 2022; Engbert & Kliegl, 2003; Hafed et al., 2011; van Ede, Chekroud, & Nobre, 2019; van Ede, Deden, & Nobre, 2021), even though they may not be obligatory for causing attention shifts (Liu, Nobre, & van Ede, 2022; Willett & Mayo, 2023). Our results showed that gaze was robustly biased towards the first-cued location regardless of whether upcoming sensory information or working-memory representations were cued.

Our study represents the first attempt to compare directly gaze biases towards external and internal stimuli within a single task. The latency of gaze biases did not differ between external and internal first cues, but the bias was more sustained when the first cue oriented attention to the external domain. The relatively smaller magnitude of the gaze bias observed after internal first cues could signify a transient access event, during which the sensory properties of the cued item are reactivated to enhance its prioritisation in memory. In contrast, when attention is cued to the location of an upcoming stimulus, it may be beneficial to sustain covert attention towards the location of the anticipated item. This pattern aligns with earlier neurophysiological research reporting alpha-power lateralisation to be sustained following pre-cues but transient following retro-cues (Mok, Myers, Wallis, & Nobre, 2016; Wallis et al., 2015). However, it is important to note that the observed pattern may not generalise to all instances of external and internal attention. In our study, external attention required spatial anticipation of an upcoming perceptual event, while in everyday situations, our attention is often focused on currently available sensory content. Similar to recent advances on a neurophysiological level (Chota, Gayet, Kenemans, Olivers, & Van der Stigchel, 2023), it would be intriguing to investigate external gaze biases when the to-be-reported sensory contents are perceptually available. This would allow for a compelling comparison against the external gaze biases observed in our present study, wherein attention was proactively guided to an upcoming sensory event.

### 4.2 Between-domain shifts suffer stronger behavioural costs

A central finding of the present study was that behaviour was more strongly impaired when shifting between as compared to within attentional domains. This is in line with results from previous literature wherein participants were required to shift within and between trials relying on perceptual information and trials relying on internal representations involving imagination (Gilbert et al., 2005), self-oriented cognition (Calzolari et al., 2022), verbal working-memory (Carlson et al., 1993; Dark, 1990; Weber, Burt, & Noll, 1986), or visual memory (Hautekiet et al., 2023; Verschooren et al., 2020; Verschooren, Liefooghe, et al., 2019). Importantly, our results reveal that the between-domain shift cost is present in continuous and dynamic behaviour drawing from short-term visual representations and sensory stimulation towards a unified behavioural response.

While RTs showed a similar between–domain shift cost for external- and internal-target reports, errors suggested that the costs of shifting between domains might depend on the direction of the shift, with larger costs when shifting from perception to working memory than vice versa. Therefore, shifting within versus between domains may have different consequences on the accessibility and quality of the to-be-reported information. The specific pattern of asymmetry we observed resembles those reported in previous research. A number of studies have demonstrated greater RT costs when transitioning from an external to an internal trials as compared to the inverse (Calzolari et al., 2022; Dark, 1990; Hautekiet et al., 2023; Verschooren et al., 2020), with some evidence for comparable asymmetries in reproduction errors (Verschooren, Liefooghe, et al., 2019). However, not all studies have shown the same directional bias (Gilbert et al., 2005). It is worth noting that these previous studies exclusively utilised binary responses instead of continuous reports, which may have limited sensitivity in detecting such asymmetries in error rates. Furthermore, direct comparisons between our study and previous research may be challenging due to differences in task demands. In particular, most previous research compared performance when shifting between trials requiring responses based on external and internal stimuli. In contrast, our study compared shifting between external and internal contents within a single trial to facilitate a unified behavioural response.

One potential explanation for the asymmetric shift cost could be attributed to memory-retrieval costs when transitioning from perception to memory. In a list-completion task, the between-domain shift cost disappeared when participants were cued about the to-be-relevant memory item 750 ms in advance, and thus could select the appropriate domain before being required to respond (Dark, 1990). Based on these findings, it was argued that the asymmetric shift cost could not purely be explained by attentional transitioning between external and internal tasks but instead must additionally reflect memory retrieval processes triggered by the internal task. Similarly, it could be argued that the asymmetrical cost seen in the reproduction errors in our study may be due to the duration required to shift attention back and retrieve information from the internal domain. However, manipulating the time between the second cue and the external display in Experiment 2 demonstrated that the asymmetric shift cost remained consistent across all tested ISIs (i.e., 0 ms, 250 ms, and 750 ms). That is, allowing for more retrieval time did not diminish or extinguish the asymmetric shift cost. Consequently, the memory retrieval account seems unlikely to explain our findings.

Alternatively, incomplete shifts and the resulting interference from the previously attended domain could have acted as a potential source of the shift-cost imbalances between domains observed in reproduction errors. Our task provided no reminders of which target to report at the response phase and instead forced participants to report whichever item was cued last before the external array. Participants may therefore have been susceptible to target confusions and biases. In our study, transitioning between as compared to within domains was more costly for internal- than external-target reports, thus, initially engaging external attention might have been more impairing on the final report than first being cued about an internal representation. Indirect evidence comes from our gaze bias findings showing that the initially cued item continued to attract attention even after the second cue was presented and this was especially pronounced if the first cue indicated an external item. However, swap rates were generally very low and did not explain the asymmetric shift cost observed in the reproduction errors. Instead, these swapping errors occurred more often for internal- than external-target reports, without being modulated by the type of shift. With this is mind, the proportion of guesses may provide a more compelling explanation for the asymmetric shift cost seen in reproduction errors, suggesting a loss of information regarding the internal display after attention was shifted to the external domain.

An alternative explanation of the shift cost asymmetries is offered by the associative interference account (Hautekiet et al., 2023; Verschooren et al., 2020; Verschooren, Liefooghe, et al., 2019), which is closely related to the Internal Dominance over External Attention hypothesis (IDEA; Verschooren & Egner, 2023). Both frameworks propose a dominant default bias towards internal as compared to external attention, resulting in more efficient shielding of internal compared to external contents. Accordingly, the processing of internal information should encounter less interference from externally available information (i.e., external-to-internal interference) compared to the reverse scenario (i.e., internal-to-external interference). Future studies that systematically investigate the nature of errors caused by both external and internal interference in either domain would go a long way in improving our understanding of interference effects and their contribution to shift-cost asymmetries.

### 4.3 Behaviour-gaze dissociations when shifting within versus between domains

Although we observed significantly slower RTs when shifting attention between as compared to within domains, directional biases in fixational gaze behaviour showed no corresponding discrepancy in the latency or magnitude following the second cue. Our gaze-bias results converge with recent research showing that microsaccade direction may not necessarily fully mimic the ultimate behavioural and neural effects of visuospatial attention (Willett & Mayo, 2023; but see van Ede, 2023). Of course, the null findings observed when contrasting gaze biases across within- and between-domain shifts should be interpreted with caution, although there are multiple factors that could explain the lack of an effect. One could for instance infer that gaze biases are a noisier measure compared to RTs and reproduction errors; however, other explanations are also possible. Gaze biases are most closely related to spatial orienting within attention, but the final behavioural performance is a cumulative measure of various attention-related functions, beyond mere orienting. Both external and internal attention involve multiple functions, such anticipating, orienting, selecting, prioritising and reprioritising, and configuring contents in preparation for upcoming response demands. These are not yet fully agreed by researchers and are likely to differ between external and internal attention by virtue of the affordances of the different domains (Myers et al., 2017; van Ede & Nobre, 2023). Our results suggest that the behavioural effects we measured at the end of the trials were not confined to the orienting functions likely proxied by gaze measures but might involve additional functions that ultimately serve as bottlenecks to performance.

For example, additional challenges can occur during the prioritisation of relevant contents after a spatial shift of attention has been performed. While the gaze bias continuously tracked spatial orienting of attention, RTs could have been delayed because it took longer to prioritise the second-cued information in between-domain shift trials. Alternatively, the difference in RTs between shift types could be due to challenges in response preparation. However, if this was the case, we would have expected the levels of shift type to be modulated by the duration of the ISI in Experiment 2. Namely, when given enough time to recover, the shift cost should have diminished. Instead, differences between shift types did not differ across ISIs, suggesting that the cognitive system was not able to recover from the detrimental effects of between-domain shifts within the tested ISIs.

**Fig. 7:**
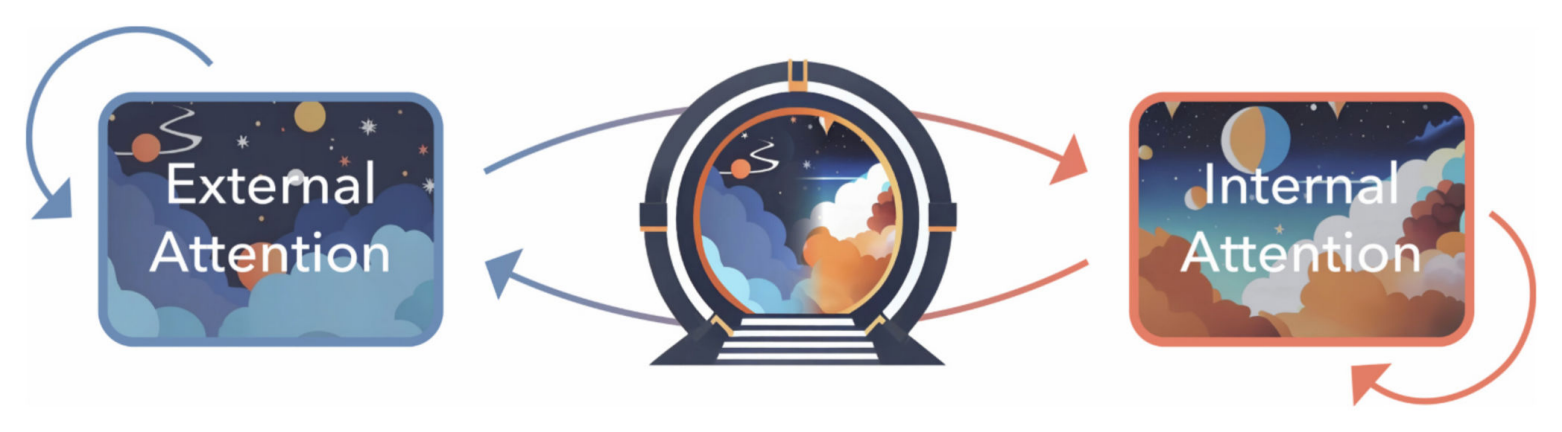
Shifts between domains might be affected by an additional control process. Our findings suggest the presence of an additional control point when shifting attention between domains, a feature not encountered during within-domain shifts. We can imagine this additional checkpoint as a portal mediating between external and internal attention.

The results of Experiment 2 suggest the presence of a distinctive aspect associated with transitioning between domains, one that extends beyond the scope of the attentional stages following orienting. This suggests the existence of a specific form of control or bottleneck that caused performance costs (cf. Burgess, Dumontheil, & Gilbert, 2007; Burgess, Gilbert, Okuda, & Simons, 2006). The processes of deactivating the currently irrelevant attentional domain and activating the relevant one may require more time compared to adjustments within the same domain, almost as if an additional switch or checkpoint occurs between these two attentional dimensions – a dynamic that is not encountered when shifting within the same domain. We can imagine this extra checkpoint as a portal between the external and internal dimensions (Fig. 7). In future studies, the utilisation of neural measures could provide valuable insights into whether there is an independent or shared control mechanism that is engaged when transitioning within and between external and internal attention.

### 4.4 Behavioural differences in reporting external and internal targets

Even though we tried to match the difficulty of external- and internal-report trials as closely as possible, our findings revealed that responses to internal targets were generally faster but less precise than responses to external targets (with the exception of the 0-ms ISI condition in Experiment 2). This discrepancy can be attributed to an inherent asymmetry between the two attentional domains in terms of their temporal dynamics and their respective competition throughout the duration of a trial. For instance, RTs in internal-report trials may have been faster because participants had an extended window for response preparation compared to external-report trials. After being cued to the internal domain, participants could select the item they would report and ignore the external display. Furthermore, the prolonged time gap between the internal display and the reporting phase may have caused some memory decay (Brown, 1958; Conrad, 1967; Cowan, 1988) and thereby contributed to greater reproduction errors when internal items were reported.

Alternatively, or additionally, interference of the stimuli within and between domains may be crucial in explaining the performance differences observed between external and internal attention. Previous literature has shown that internal representations can be biased and disrupted by external interference (Gresch, Boettcher, van Ede, & Nobre, 2021; Lorenc, Mallett, & Lewis-Peacock, 2021; Rademaker, Bloem, De Weerd, & Sack, 2015; Teng & Kravitz, 2019; van Ede, Board, & Nobre, 2020) as well as by other memory representations (Bae & Luck, 2017; Brady & Alvarez, 2011; Chunharas, Rademaker, Brady, & Serences, 2022; Dubé, Zhou, Kahana, & Sekuler, 2014; Huang & Sekuler, 2010; Teng & Kravitz, 2019). These sources of interference could therefore have exerted a more pronounced influence on internal as opposed to external contents. In line with that, recent research has demonstrated that the influence of internal representations on perception decreases with sufficient time between an encoding display and a perceptual display (i.e., from 550 ms onwards), while internal representations become increasingly biased by perception (Teng, Kaplan, Shomstein, & Kravitz, 2023). Therefore, in the present study, as the memory stimuli recede into the past, they might have become less distinguishable from the external stimulation, increasing the overall swap rate in internal- as compared to the external-target trials. Systematically varying the time interval between the first and second cue within our study design could yield valuable insights into the dynamic interplay of factors causing performance differences between external and internal attention.

Furthermore, the necessity to perform a shift of attention, especially following the cueing of another item, may have exerted a more pronounced influence on internal- as opposed to external-target reports. Retro-cue studies have demonstrated that reorienting attention within the internal domain can sometimes be associated with a cost for the uncued internal representation (Griffin & Nobre, 2003; Pertzov, Bays, Joseph, & Husain, 2013), although not all studies have consistently demonstrated a performance decrement for the uncued item (Li & Saiki, 2014; Lin & Fougnie, 2022; Myers, Chekroud, Stokes, & Nobre, 2018; Rerko & Oberauer, 2013). Moreover, in external-to-internal trials, being initially cued about an external item may have impaired the rehearsal of memory items, thereby potentially further compromising working-memory performance (Awh, Jonides, & Reuter-Lorenz, 1998; Johnson & Spencer, 2016). In contrast, shifts within the external attentional domain do not appear to exert a significant impact on external-task performance (Dowd & Golomb, 2019). In future research, it is crucial to delve more directly into the impact of attentional reorienting in perception and working memory on subsequent behaviour.

While a naturalistic study of shifting attention between domains necessitates a dynamic task setting, we recognise that various factors may have contributed to the performative differences between reporting items based on perception and working memory. In the current study, we can only highlight and offer speculative insights into the potential roles of these factors. Future integration of neural measures will prove invaluable in disentangling their respective contributions to the observed effects.

## 5 Conclusion

Our two experiments open new avenues for investigating the fundamental property of shifting attention between perception and working memory. We revealed that shifting between as compared to within domains incurs behavioural costs and that these costs persisted even when additional time was given to complete the shift. Gaze biases, acting as a proxy of spatial orienting functions, did not mirror the behavioural performance patterns, suggesting bottlenecks at additional attention functions. To shed light on these mechanisms, future research should utilise techniques with high temporal resolution, such as magnetencephalography (MEG), to compare the neural processes and dynamics underlying within- and between-domain shifts. This will enable a more comprehensive understanding of the cognitive and neural mechanisms involved in this ubiquitous act of shifting attention between the external environment and working memory to fulfil goals in everyday cognition.

## Data Availability

Experimental code, data, and analysis scripts can be found online at hhttps://osf.io/mcexr/.

## Footnotes

1 We will use the term ‘cued item’ to refer to an external location where an upcoming bar is going to appear (in perception) as well as to an internal visual representation of a bar (in working memory).

**Appendix A. Supplementary data**
Supplementary data to this article can be found online at https://doi.org/10.1016/j.cognition.2024.105731.
